# Longitudinal transitions in initiation, cessation, and relapse of cigarette smoking and e-cigarette use among US youth and adults: Validation of a microsimulation model

**DOI:** 10.1371/journal.pone.0284426

**Published:** 2023-04-14

**Authors:** Eli Schwamm, Farzad Noubary, Nancy A. Rigotti, Krishna P. Reddy

**Affiliations:** 1 Medical Practice Evaluation Center, Mongan Institute, Massachusetts General Hospital, Boston, Massachusetts, United States of America; 2 Harvard Medical School, Boston, Massachusetts, United States of America; 3 Bouvé College of Health Sciences, Northeastern University, Boston, Massachusetts, United States of America; 4 Tobacco Research and Treatment Center, Mongan Institute, Massachusetts General Hospital, Boston, Massachusetts, United States of America; 5 Division of General Internal Medicine, Massachusetts General Hospital, Boston, Massachusetts, United States of America; 6 Division of Pulmonary and Critical Care Medicine, Massachusetts General Hospital, Boston, Massachusetts, United States of America; Public Library of Science, UNITED KINGDOM

## Abstract

**Introduction:**

Estimates of initiation, cessation, and relapse rates of tobacco cigarette smoking and e-cigarette use can facilitate projections of longer-term impact of their use. We aimed to derive transition rates and apply them to validate a microsimulation model of tobacco that newly incorporated e-cigarettes.

**Methods:**

We fit a Markov multi-state model (MMSM) for participants in Waves 1–4.5 of the Population Assessment of Tobacco and Health (PATH) longitudinal study. The MMSM had nine cigarette smoking and e-cigarette use states (current/former/never use of each), 27 transitions, two sex categories, and four age categories (youth: 12-17y; adults: 18-24y/25-44y/≥45y). We estimated transition hazard rates, including initiation, cessation, and relapse. We then validated the Simulation of Tobacco and Nicotine Outcomes and Policy (STOP) microsimulation model, by: (a) using transition hazard rates derived from PATH Waves 1–4.5 as inputs, and (b) comparing STOP-projected prevalence of smoking and e-cigarette use at 12 and 24 months to empirical data from PATH Waves 3 and 4. We compared the goodness-of-fit of validations with “static relapse” and “time-variant relapse,” wherein relapse rates did not or did depend on abstinence duration.

**Results:**

Per the MMSM, youth smoking and e-cigarette use was generally more volatile (lower probability of maintaining the same e-cigarette use status over time) than that of adults. Root-mean-squared error (RMSE) for STOP-projected versus empirical prevalence of smoking and e-cigarette use was <0.7% for both static and time-variant relapse simulations, with similar goodness-of-fit (static relapse: RMSE 0.69%, CI 0.38–0.99%; time-variant relapse: RMSE 0.65%, CI 0.42–0.87%). PATH empirical estimates of prevalence of smoking and e-cigarette use were mostly within the margins of error estimated by both simulations.

**Discussion:**

A microsimulation model incorporating smoking and e-cigarette use transition rates from a MMSM accurately projected downstream prevalence of product use. The microsimulation model structure and parameters provide a foundation for estimating the behavioral and clinical impact of tobacco and e-cigarette policies.

## Introduction

Electronic cigarettes (e-cigs) are battery-operated nicotine delivery devices that produce an aerosol for inhalation (“vaping”) but do not burn tobacco. There has been a dramatic increase in the use of e-cigs in the last decade in the US, sparking debates about their potential harms and benefits. E-cig use among US high school students rose from 1.5% in 2011 to 27.5% in 2019, raising concern about a youth vaping epidemic and the potential for nicotine addiction or transition to cigarette smoking among those who otherwise might not use tobacco or nicotine products [1, 2]. Conversely, several clinical trials and observational studies have shown that e-cig use can help adults to quit combustible cigarette smoking, and some see e-cig use as a potential harm reduction strategy [3–6]. Meanwhile, in 2019, in response to the increased use of e-cigs among youth and the cases of e-cigarette or vaping product use associated acute lung injury (EVALI), several states and municipalities and the federal government prohibited the sale of non-tobacco flavored or all e-cigs [7–9]. In this context, plus that of the COVID-19 pandemic, e-cig use among US high school students decreased to 11.3% in 2021, though changes in survey methodology limit direct comparisons with data from prior surveys [10]. However, the net public health impact of e-cig policies, in terms of their influence on youth uptake, smoking cessation or reduction among adults, and direct toxicity, remains unclear. Simulation models provide a useful framework for evaluating downstream outcomes of policies.

To inform simulation models, estimates of transition rates between various states of cigarette smoking, e-cig use, and dual use–including initiation, cessation, and relapse–are needed. These estimates, themselves, may also illuminate associations between e-cig use and smoking initiation, cessation, and relapse. Transition modeling is increasingly being used to evaluate secular trends and policy impacts. There are some published statistical models of tobacco and nicotine product transition rates [11–16]. However, for detailed microsimulation models, individual-level transition estimates stratified by various key categories, including youth/adults, never/former/current use, and tobacco smoking/e-cig/dual use, are needed.

We sought to estimate individual-level behavioral transitions, among both youth and adults, between each combination of current, former, and never smoking and e-cig use states. We also aimed to validate a microsimulation model of tobacco smoking and e-cig use using these transition estimates as input parameters, determining how accurately the transition estimates would project the subsequent prevalence of smoking and e-cig use. If validated, individual-level behavioral transitions can be used in simulation models to project longer-term clinical and economic outcomes associated with smoking, e-cig use, and associated regulatory policies, which is an eventual goal of this methodologic development.

## Methods

### Data source and statistical tools

**Fig 1:**
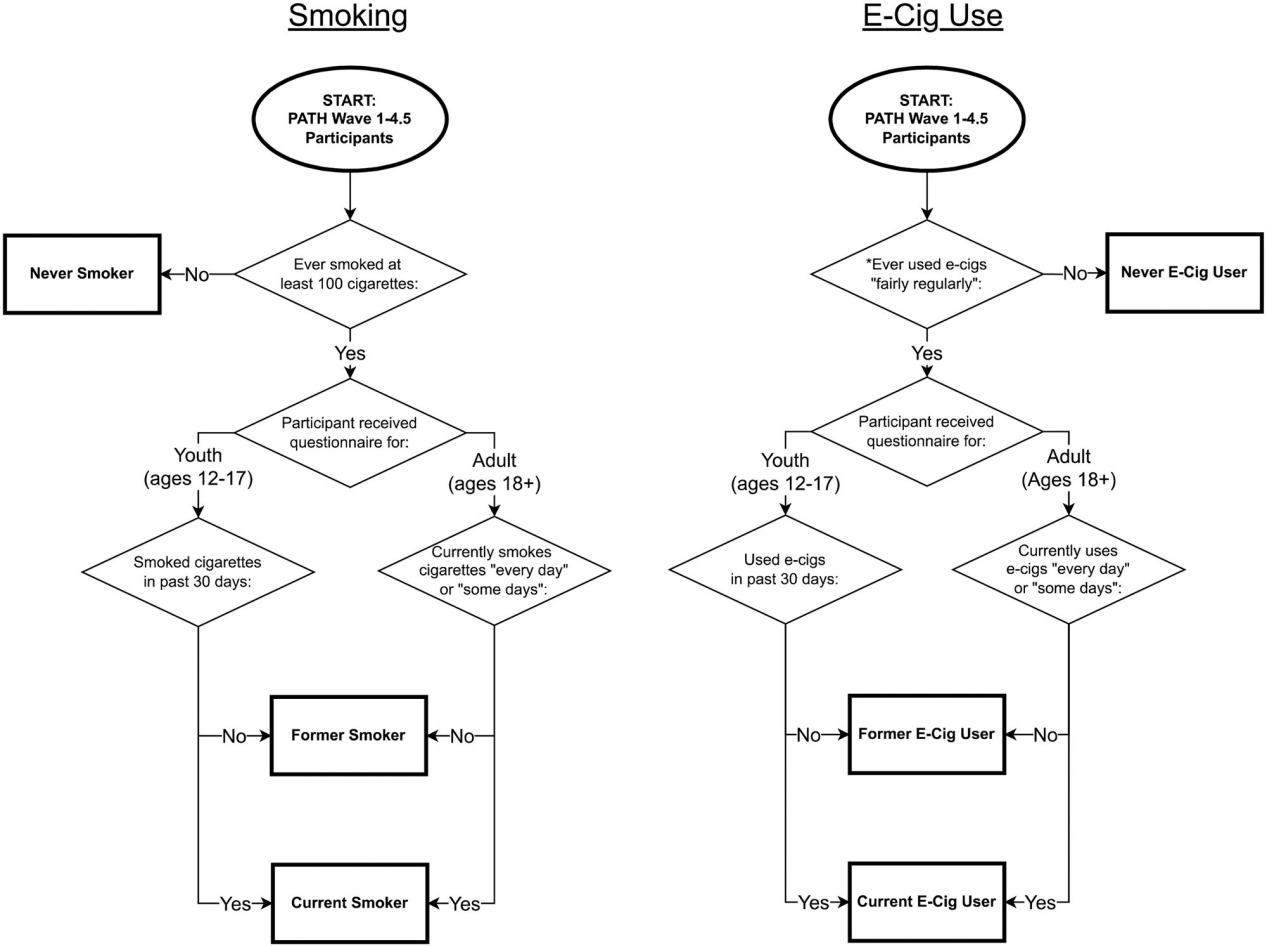
Classification of survey participants as never, former, and current smokers/e-cig users. Population Assessment of Tobacco and Health (PATH) Study Waves 1–4.5 respondents were classified into never, former, and current smoking/e-cig use. A single participant’s classification could differ between the two products: e.g., former smoker/current e-cig user. In youth survey Waves 1–2 and adult survey Wave 1, regarding electronic products, participants were asked only about “electronic-cigarettes.” In all other survey waves, participants were asked about e-product use, which included "e-cigarettes, vape pens, personal vaporizers and mods, e-cigars, e-pipes, e-hookah or hookah pens." For this analysis, we considered an affirmative response to either of these questions as indicative of e-cig use. *Youth in Wave 1 were asked to attest to ever use instead of ever “fairly regular” use of e-cigs. In our analysis, youth in Wave 1 who attested to never having used e-cigs were classified as never e-cig users. We classified all other youth as “undefined” for Wave 1 e-cig use and excluded them from the analysis in that wave.

We analyzed transitions in cigarette smoking and e-cig (e-cigarette, vape pen, personal vaporizer and mods, e-cigar, e-pipe, e-hookah or hookah pen) use in the Population Assessment of Tobacco and Health (PATH) Study, a nationally representative longitudinal survey of the US noninstitutionalized population ages 12 years and older [17–19]. Wave 1 of PATH includes survey responses from 13,500 youth (ages 12–17 years) and 32,000 adults (ages ≥18 years) from September 2013 to December 2014. An additional 7,000 youth ages 9–11 years (household members of other respondents) were identified during the Wave 1 sampling process and were surveyed in subsequent waves after reaching 12 years of age. As of May 2021, survey responses were also available from Wave 2 (October 2014 to October 2015, youth and adults), Wave 3 (October 2015 to October 2016, youth and adults), Wave 4 (December 2016 to January 2018, youth and adults), and Wave 4.5 (December 2017 to December 2018, youth only). All PATH questionnaires evaluate cigarette and e-cig product use, but questions vary between youth and adults and by wave (Fig 1).

The PATH data include weights to adjust for bias introduced by complex survey design and non-response. We weighted responses with Wave 4 and 4.5 longitudinal weights, as recommended by the PATH investigators [20]. We accounted for aging of adolescents into the adult cohort, incorporating all aged-up adolescents who had Wave 4 longitudinal weights–i.e., all except those who were lost to follow-up at Wave 4. We included in the analysis all youth and adults with complete survey responses in Waves 4.5 and 4, respectively, and at least one prior wave, amounting to 7,808 youth and 21,844 adults (Table A in S1 File). We conducted analyses in R version 3.6.3 with packages “tidyverse” version 1.3.0 and “msm” version 1.6.8.

### Definitions of smoking and e-cig use status

We merged data across youth and adult questionnaire responses from Waves 1–4.5 and classified participants as current, former, or never cigarette smokers, and as current, former, or never e-cig users (Fig 1). We considered participants to be established smokers/e-cig users if they had smoked over 100 cigarettes in their lifetime or ever used e-cigs “fairly regularly”, respectively [21, 22]. Among established smokers/e-cig users, we distinguished current from former smoking/e-cig use by past 30-day use for youth and current every-day or some-day use for adults. Participants with no established smoking/e-cig use were classified as never smokers/e-cig users. We excluded survey responses where smoking and e-cig use data were missing, unless participant use status could be determined deductively (i.e., they were classified as a never smoker/never e-cig user in a future wave).

### Continuous time Markov multi-state model

**Fig 2:**
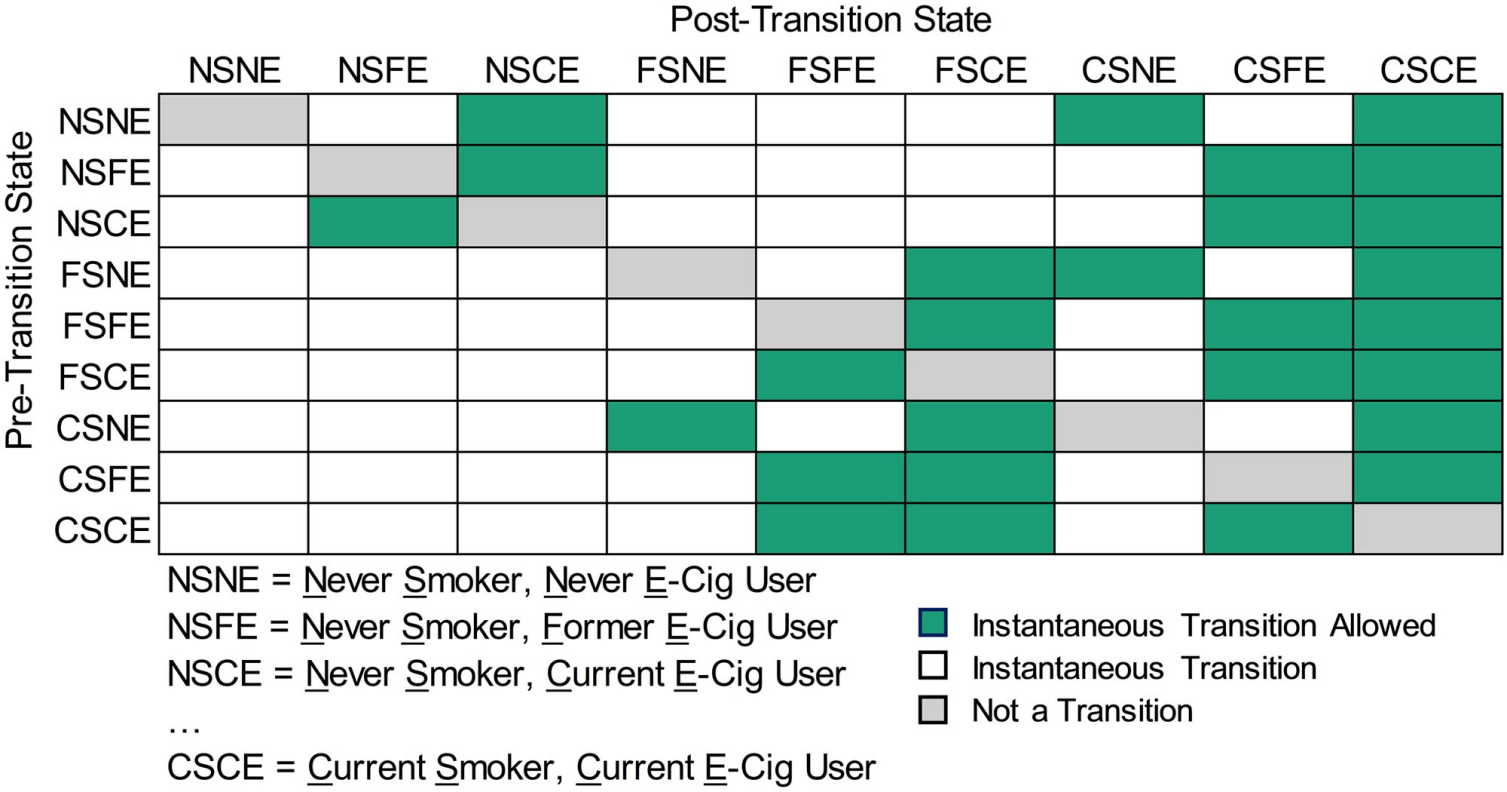
Allowed and disallowed instantaneous transitions in the continuous-time Markov multi-state model of cigarette smoking and e-cig use states. Continuous time Markov multi-state models do not require exact transition times to be observed and allow multiple transitions to occur between observations. They therefore require allowed instantaneous transitions to be specified. Allowed instantaneous transitions from the indicated pre-transition smoking and e-cig use state (row) to the indicated post-transition state (column) are in green. Disallowed instantaneous transitions are in white. We disallowed transitions that would entail never users going directly to a former use state and those that would entail former users or current users going to a never use state. Cells in gray are those reflecting staying in the same state.

Continuous time Markov multi-state models are suitable for estimating, from longitudinal data, the hazard rates at which individuals transition between pre-determined states. These models do not require exact transition times to be observed and allow multiple transitions to occur consecutively between observations, or consecutively over several observations [23]. In this analysis, we defined nine states corresponding to the combinations of: (1) current, former, and never cigarette smoking, and (2) current, former, and never e-cig use. We specified 27 allowable instantaneous state-transitions (Fig 2 and Supplemental Methods in S1 File). We incorporated age category at each survey wave (12–17, 18–24, 25–44, ≥45 years) and sex (female or male) as covariates. We incorporated survey weights into transition hazard rate estimates (Supplemental Methods in S1 File). We included all the earlier PATH waves to increase the number of observed transitions and enable parametrization of the model, including by age and sex. Many transitions, such as smoking initiation, occur rarely, and limiting the analysis to only two waves would result in an insufficient number of transitions to parametrize the model.

### Transition hazard rates and annual cumulative transition probabilities

The multi-state model directly estimates transition hazard rates, which measure the instantaneous likelihood of each allowable transition. Annual cumulative transition probabilities, also derived from hazard rates, estimate the proportion of people in a prior state expected to be in a subsequent state one year later, where some individuals will undergo multiple transitions within the year [23]. We present both transition hazard rates and annual cumulative transition probabilities.

### Validation of a tobacco and nicotine outcomes and policy microsimulation model

The Simulation of Tobacco and Nicotine Outcomes and Policy (STOP) microsimulation model includes age- and sex-stratified probabilities of tobacco and nicotine product initiation, cessation, and relapse. We previously validated the STOP model with tobacco smoking data and mortality data from the National Health Interview Survey and the linked National Death Index [24]. Subsequently, we updated the model to incorporate e-cig use, specifically adding states of current/former/never e-cig use and dual product use. Here, we parametrized the STOP model with our newly derived transition hazard rate estimates (converting them from annual to monthly rates and then to monthly probabilities for use in the simulation model) and evaluated how accurately the STOP model could predict prevalence of each of the nine smoking and e-cig use states over time (Supplemental Methods in S1 File).

We populated STOP’s initial age, sex, and cigarette smoking and e-cig use distributions with the corresponding estimates at PATH Wave 2 (PATH Wave 1 lacked the necessary questions to distinguish between established and experimental e-cig use among youth, Fig 1). We ran the STOP model for 24 months, extracted model-projected prevalence of smoking and e-cig use for the entire population at each month, and compared model-projected prevalence at 12 months and at 24 months to empirical data from PATH Waves 3 and 4 [11, 24, 25]. Participants who were younger than 12 years old at Wave 2, and therefore were not included in the STOP simulated cohort, were excluded from PATH Waves 3 and 4 empirical prevalence estimates. We assessed goodness of model fit via root-mean-squared error (RMSE) between projected (STOP model) and empirical (PATH data) prevalence.

### Validation of time-variant relapse

Continuous time Markov multi-state models make the simplifying assumption that transition hazard rates do not depend on the duration spent in the current state (e.g., that a current smoker of one year and a current smoker of 10 years have equal rates of smoking cessation). However, smoking relapse probabilities are known to depend on duration of abstinence [26, 27]. It is plausible that e-cig relapse probabilities follow a similar pattern, given that nicotine is the common addictive drug in both cigarettes and e-cigs. The STOP model allows rates of smoking and e-cig relapse to decay exponentially with respect to the number of months of abstinence (Supplemental Methods in S1 File). We performed a second, “time-variant relapse” validation in which we utilized this feature over a 24-month time horizon and compared the results to both empirical estimates from PATH and the previously described static relapse validation.

### Ethics statement

The protocol for this study was approved by the Mass General Brigham Institutional Review Board, which waived the need for informed consent as all data were fully anonymized and publicly available.

## Results

### Characteristics of PATH data

The weighted PATH survey response rate among adults in Waves 1–4 was 73.5% and among youth in Waves 1–4.5 was 74.6% [20]. Among those who responded to the survey, the weighted response rate for age category was >99.9%, for sex was 99.9%, and for smoking and e-cig use state was 91.2%. Participants completed a weighted median of 4 survey waves. Table A in S1 File indicates smoking and e-cig use prevalence by wave, age, and sex.

### Transitions in cigarette smoking and e-cig use

**Fig 3:**
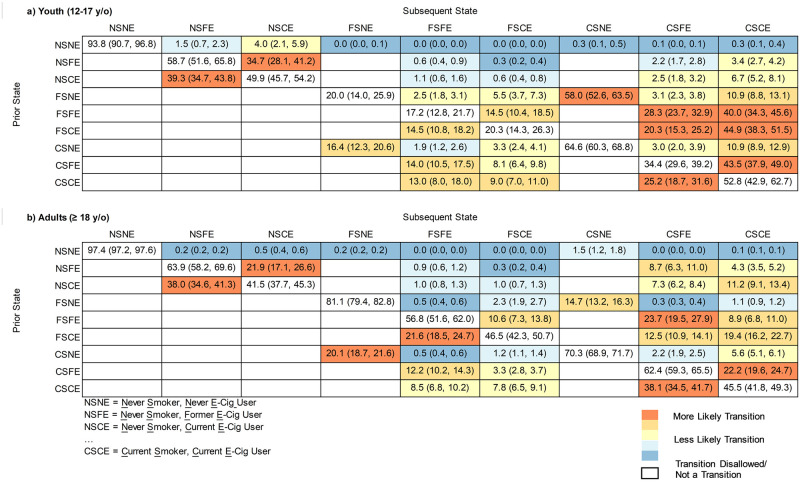
Annual cumulative transition probabilities according to the continuous-time Markov multi-state model for (a) youth and (b) adults. Data are presented as the probability, among those in the indicated prior state (row), of being in the indicated subsequent state (column) one year later. In parentheses are the 95% confidence intervals. More likely transitions are pictured in orange, while less likely transitions are pictured in blue. Note that participants may experience more than one instantaneous transition in sequence within the year, meaning that some transitions that cannot occur instantaneously (e.g., NSNE to FSFE) are nonetheless allowed as annual cumulative transitions.

Table 1 indicates PATH-derived hazard rates for each of the transitions in cigarette smoking and e-cig use for females ages 12–17 years, as well as the transition hazard rate ratios for different age groups (18–24 years, 25–44 years, and ≥45 years) and for males compared with females. Fig 3 indicates annual cumulative transition probabilities between various states of cigarette smoking and e-cig use among youth (panel a) and adults (panel b). Table B in S1 File shows the number of unique transitions in youth and in adults (unweighted). Youth smoking and e-cig use was generally more volatile (lower probability of maintaining the same smoking and e-cig use status over time) than that of adults, even when restricting the latter group to those aged 18–24 years (Table 1). In general, age had a greater influence than sex on transition rates (Table 1). Of relevance to the ongoing public health debate around e-cigs: among youth never smokers, there was substantial volatility in e-cig use but relatively little initiation of cigarette smoking; among adult current smokers, there was more volatility in e-cig use than in cigarette smoking; and among adult former smokers, e-cig use was less volatile than or similarly volatile as cigarette smoking.

**Table 1:** Continuous time Markov model-estimated baseline transition hazard rates and adjusted hazard rate ratios.

Transition	Transition Hazard Rate: Baseline, 12 to 17 years old, Female, Rate per 100 Person-Years (95% CI)	Transition Hazard Rate Ratio: 18 to 24 years old, Female, Ratio (95% CI)	Transition Hazard Rate Ratio: 25 to 44 years old, Female, Ratio (95% CI)	Transition Hazard Rate Ratio: ≥ 45 years old, Female, Ratio (95% CI)	Transition Hazard Rate Ratio: Male Ratio (95% CI)
NSNE to NSCE	4.57 (2.50, 8.34)	1.19 (0.86, 1.64)	0.17 (0.09, 0.31)	0.07 (0.03, 0.16)	1.79 (1.54, 2.08)
NSNE to CSNE	0.33 (0.18, 0.62)	4.14 (2.56, 6.67)	3.67 (2.27, 5.93)	5.46 (3.46, 8.60)	1.44 (1.24, 1.67)
NSNE to CSCE	< 0.00 (< 0.00, < 0.00)	1.01 (0.85, 1.21)	0.82 (0.64, 1.05)	2.42 (1.65, 3.55)	1.53 (1.23, 1.90)
NSFE to NSCE	79.47 (54.81, 115.22)	0.48 (0.36, 0.64)	0.38 (0.24, 0.58)	0.89 (0.65, 1.21)	0.96 (0.71, 1.31)
NSFE to CSFE	4.35 (3.12, 6.07)	1.09 (0.71, 1.67)	1.40 (1.09, 1.80)	5.95 (4.24, 8.35)	1.10 (0.86, 1.41)
NSFE to CSCE	0.03 (0.00, 80.85)	0.81 (0.66, 0.99)	0.54 (0.44, 0.67)	1.90 (1.56, 2.31)	1.11 (0.88, 1.41)
NSCE to NSFE	93.20 (75.80, 114.60)	1.12 (0.92, 1.38)	1.07 (0.82, 1.39)	0.82 (0.61, 1.09)	0.90 (0.75, 1.07)
NSCE to CSFE	0.01 (0.00, 18.24)	0.65 (0.50, 0.85)	1.89 (1.46, 2.43)	3.49 (2.68, 4.56)	0.99 (0.65, 1.49)
NSCE to CSCE	12.00 (9.36, 15.37)	1.23 (0.90, 1.69)	1.10 (0.83, 1.45)	3.07 (2.27, 4.16)	1.40 (1.13, 1.72)
FSNE to FSCE	25.26 (18.89, 33.78)	1.00 (0.74, 1.36)	0.21 (0.16, 0.27)	0.05 (0.03, 0.07)	1.52 (0.96, 2.40)
FSNE to CSNE	249.81 (192.32, 324.49)	0.57 (0.47, 0.69)	0.15 (0.13, 0.19)	0.04 (0.03, 0.05)	0.81 (0.65, 1.00)
FSNE to CSCE	0.13 (0.00, 89.85)	0.48 (0.39, 0.59)	0.51 (0.42, 0.62)	0.47 (0.37, 0.60)	0.21 (0.15, 0.30)
FSFE to FSCE	104.65 (70.10, 156.22)	0.34 (0.26, 0.44)	0.30 (0.23, 0.38)	0.12 (0.09, 0.16)	1.04 (0.76, 1.42)
FSFE to CSFE	155.29 (113.69, 212.11)	0.44 (0.36, 0.54)	0.27 (0.21, 0.36)	0.24 (0.17, 0.33)	0.89 (0.66, 1.21)
FSFE to CSCE	25.25 (11.06, 57.65)	1.25 (0.88, 1.78)	0.15 (0.12, 0.19)	0.05 (0.04, 0.06)	3.91 (3.03, 5.05)
FSCE to FSFE	71.43 (53.77, 94.90)	1.21 (0.92, 1.58)	0.77 (0.61, 0.96)	0.42 (0.34, 0.53)	0.93 (0.72, 1.21)
FSCE to CSFE	10.12 (5.56, 18.40)	13.88 (11.39, 16.92)	0.18 (0.14, 0.23)	0.16 (0.09, 0.29)	0.14 (0.10, 0.20)
FSCE to CSCE	125.53 (90.13, 174.84)	0.49 (0.38, 0.62)	0.38 (0.28, 0.52)	0.23 (0.17, 0.30)	1.26 (0.92, 1.74)
CSNE to FSNE	65.70 (53.16, 81.19)	0.56 (0.48, 0.66)	0.38 (0.32, 0.44)	0.43 (0.36, 0.51)	0.94 (0.82, 1.08)
CSNE to FSCE	0.86 (0.50, 1.48)	1.14 (0.91, 1.43)	1.10 (0.81, 1.49)	0.58 (0.46, 0.75)	1.69 (1.29, 2.21)
CSNE to CSCE	18.13 (15.21, 21.62)	1.23 (1.07, 1.42)	0.75 (0.66, 0.86)	0.36 (0.31, 0.42)	1.01 (0.89, 1.15)
CSFE to FSFE	55.55 (42.31, 72.94)	0.55 (0.43, 0.70)	0.35 (0.27, 0.46)	0.29 (0.23, 0.36)	1.14 (0.87, 1.49)
CSFE to FSCE	0.61 (0.20, 1.93)	0.37 (0.28, 0.47)	0.82 (0.61, 1.11)	2.70 (2.02, 3.61)	1.09 (0.87, 1.37)
CSFE to CSCE	139.16 (111.21, 174.13)	0.53 (0.43, 0.65)	0.44 (0.35, 0.55)	0.30 (0.24, 0.37)	0.80 (0.61, 1.03)
CSCE to FSFE	22.53 (9.89, 51.33)	0.27 (0.18, 0.39)	0.07 (0.06, 0.10)	0.26 (0.15, 0.46)	3.11 (2.34, 4.14)
CSCE to FSCE	15.84 (11.91, 21.06)	2.05 (1.64, 2.56)	1.16 (0.90, 1.50)	0.75 (0.59, 0.95)	1.09 (0.84, 1.41)
CSCE to CSFE	64.03 (41.82, 98.02)	1.26 (0.96, 1.65)	1.29 (0.93, 1.80)	1.23 (0.88, 1.71)	0.97 (0.82, 1.13)

Transition hazard rate ratios, adjusted by sex (female or male) and age category (12–17, 18–24, 25–44, ≥45 years), are juxtaposed against baseline transition rates for female youth (ages 12–17 years). To estimate a transition rate for any subgroup, the baseline rate is multiplied by the corresponding subgroup rate ratio(s). E.g., men ages 18–24 transition from NSNE to NSCE at a rate of 4.57*1.19*1.79 = 9.73 per 100 person-years.

NSNE = Never Smoker, Never E-Cig User

NSFE = Never Smoker, Former E-Cig User

NSCE = Never Smoker, Current E-Cig User

CSCE = Current Smoker, Current E-Cig User

### Validation of transition estimates: Static relapse and time-variant relapse

We applied the newly derived transition probabilities (converted from hazard rates) as input parameters in the STOP model and compared STOP model output for cigarette smoking prevalence and e-cig use prevalence to that reported in subsequent waves of PATH. Root-mean-squared error for model-projected versus empirical prevalence of smoking and e-cig use was less than 0.7% for both the static relapse and time-variant relapse STOP simulations, with no significant difference in goodness-of-fit between the two (for static relapse: RMSE 0.69%, 95% confidence interval [CI] 0.38–0.99%; for time-variant relapse: RMSE 0.65%, CI 0.42–0.87%). The largest contributors of error were, in the static relapse simulation, the proportion of former smoker/never e-cig users at Wave 4 (projected prevalence 20.6% vs. empirical prevalence 22.7%; difference of 2.1%, CI 1.0–3.2%), and, in the time-variant relapse simulation, the proportion of former smoker/never e-cig users at Wave 3 (projected prevalence 20.1% vs. empirical prevalence 21.8%; difference of 1.7%, CI 0.7–2.7%). Empirical estimates of prevalence of smoking and e-cig use from PATH were mostly within the margin of error estimated by both static relapse and time-variant relapse simulations (Table C and Fig C in S1 File).

## Discussion

We leveraged a continuous time Markov multi-state model to estimate rates of cigarette smoking and e-cig initiation, cessation, and relapse using longitudinal data from PATH Waves 1–4.5, incorporating age and sex as covariates. The estimated first transition probabilities were similar to those estimated by a previously published Markov multi-state model of adults, though differences in model structure only allow direct comparison among adult never smoker/never e-cig users and adult current smoker/current e-cig users, and there are differences between the two studies in definitions of current use [11]. We used our estimates, including those for youth, to validate the STOP microsimulation model, which projected prevalence of cigarette smoking and e-cig use over time that were similar to the empirical prevalence in PATH Wave 3 and Wave 4.

Microsimulation models, like STOP, are important tools for projecting tobacco and e-cig use behavior and the downstream health effects, including clinical and health economic consequences. External factors, such as policies intended to influence tobacco and e-cig use, can be introduced into microsimulation models, thereby providing decision makers with estimates of the potential impact of these policies. However, to have confidence in the projections of microsimulation models, robust estimates of background tobacco and e-cig transition rates that account for both current and past use of tobacco cigarettes and e-cigs are needed. Differentiating between youth and adults in these estimates is critical–for example, a policy (e.g., a tax) that aims to curb e-cig use may be beneficial for youth public health but harmful for adult public health if it deters adult cigarette smokers from switching to a potentially less harmful product.

This analysis includes several aspects chosen to maximize its utility in parametrizing a tobacco and nicotine policy microsimulation model. First, as per PATH definitions, we focused on established rather than experimental cigarette smoking and e-cig use, which better aligns with longer-term health risks [12, 13, 28]. Second, our modeling of all nine combinations of current, former, and never smoking/e-cig use allows future work to ascribe distinct disease incidence and mortality risks (e.g., myocardial infarction, lung cancer) to each use state. For example, never smoker/current or former e-cig users have different morbidity and mortality risks over time compared with former smoker/current or former e-cig users [28]. Third, our exploration of relapse rates that decay exponentially with respect to duration of abstinence reflects the reality that most quit attempts result in early relapse and that a longer duration of abstinence is associated with a lower probability of relapse [26, 27]. Interestingly, we did not find convincing evidence that static relapse rates differ from time-variant relapse rates in terms of RMSE in their ability to predict PATH Wave 3 and Wave 4 prevalence of smoking and e-cig use. It is possible that the duration of follow-up for those who quit smoking or e-cig use in PATH Waves 1–4 was insufficient to reveal major differences in these two microsimulations. Longer-term simulations might reveal more substantial differences between the two approaches. Fourth, our simultaneous analysis of both youth and adults allows some comparisons between the two groups. We found that smoking and e-cig use were generally more volatile among youth than adults, even when restricting the comparison to youth versus young adults aged 18–24 years. Quantifying such differences facilitates future analysis to capture any differential impact among youth and adults of policy interventions around cigarette smoking and e-cig use [11–13, 16].

Youth e-cig uptake itself is noteworthy. We found that youth never smoker/never e-cig users were less likely than their adult counterparts to start smoking but more likely to start using e-cigs. Youth current e-cig users were very likely to quit, but equally likely to subsequently restart e-cig use even if they did quit, a dynamic that can only be captured by longitudinal data such as those in the PATH Study. Further study is warranted to investigate if this instability is indicative of casual use that is dependent upon social context (e.g., youth who start and stop e-cig use based on the behavior of their peers) or is indicative of nicotine addiction and difficulty maintaining abstinence after cessation attempts.

This study cannot determine causal relationships. However, it adds nuance to the evidence of the associations between e-cig use and smoking transitions. In contrast to prior studies, we separated never smoker/current and former e-cig users from former smoker/current and former e-cig users, and could therefore limit the confounding associated with smoking history [11–13].

As the PATH Study collects additional waves of data, future models may incorporate the number of previous quit attempts and other sociodemographic variables as covariates to adjust for confounding. We accounted for age and sex. The magnitude and frequency of meaningful differences between transition rates was greater when comparing youth and adults than when comparing women and men.

This work has several limitations. The majority of observations in PATH Waves 1–4.5 predate the rapid rise of pod-based e-cigs such as JUUL in late 2017 and 2018, the EVALI reports in 2019–2020, and the COVID-19 pandemic that emerged in the US in 2020. Considering the higher nicotine content and more rapid nicotine absorption of JUUL products relative to other e-cigs, and the collateral consequences of the EVALI outbreak and the COVID-19 pandemic, it is plausible that the dynamics of smoking and e-cig use may have changed since 2018. Further, it is plausible that transition rates, such as the rate of e-cig initiation among e-cig naïve youth, may have changed over the course of Waves 1–4.5 in the context of time-varying regulatory policies and market changes. However, results of the internal validation suggest that such a change is not significant enough to reduce the predictive power of our model. Our validation exercise was limited by dependence on a subset of PATH data as the validation set. Questions about tobacco smoking and e-cig use differ across cohort studies, complicating comparisons especially of dual use states and limiting the ability to perform meaningful independent external validation.

Our estimates account for age and sex but not for race, socio-economic status, level of nicotine dependence, household member and peer use of cigarettes and e-cigs, exposure to product advertising, and local tobacco regulatory policies, which may be associated with differences in cigarette smoking and e-cig use behaviors. Our inclusion of many states and transitions made the computational intensity of including such variables infeasible. Nonetheless, our results provide background age- and sex-based transitions, from nationally representative PATH data, that can be used to evaluate the overall impact of policies. Additionally, our estimates can be integrated with race/ethnicity, education, and income hazard ratios estimated by others, in models with fewer states and transitions, to project smoking and e-cig use stratified by these characteristics [11]. Our definition of current and former smoking or e-cig use among youth includes only established use, different from the experimental use included in reports by the Centers for Disease Control and Prevention. If we were to choose different definitions of current use between youth (established or experimental) and adults (established only), there would be youth current smokers or current e-cig users who transition to never smokers or never e-cig users when they age into the adult cohort. Our model does not allow for these transitions. Excluding experimental use is more consistent with future aims of the STOP model to predict the health outcomes associated with longer-term smoking and e-cig use.

In summary, we used longitudinal data around tobacco cigarette smoking and e-cig use among youth and adults from PATH Waves 1–4.5 to develop a continuous-time Markov multi-state model with nine cigarette smoking and e-cig use states, 27 allowable instantaneous transitions, two sex categories, and four age categories. We subsequently validated a tobacco and e-cig outcomes and policy microsimulation model parametrized with these newly derived transition estimates. The microsimulation model predicted prevalence of cigarette smoking and e-cig use over a 2-year time horizon with RMSE <0.7% compared with empirical data. In future work, simulation models can be populated with these estimates along with additional data on smoking and e-cig use-stratified mortality, noncommunicable disease incidence, quality of life, and health care costs to predict the outcomes and cost-effectiveness of proposed policy interventions targeting smoking and e-cig use [24].

## Supporting information

S1 File Supporting information. (DOCX)
