# Supplementary material for: Longitudinal transitions in initiation, cessation, and relapse of cigarette smoking and e-cigarette use among US youth and adults: Validation of a microsimulation model
Source: PLoS One. 2023 Apr 14;18(4):e0284426. doi: 10.1371/journal.pone.0284426 (PMC10104340; doi:10.1371/journal.pone.0284426)
Supplement: S1 File — (DOCX) [file pone.0284426.s001.docx]

**Supporting Information**

**Longitudinal transitions in initiation, cessation, and relapse of tobacco smoking and**

**e-cigarette use among US youth and adults: Validation of a microsimulation model**

**Table of Contents**

[**Supplemental methods** 2](#_Toc126599540)

[**Adding survey weights to the MSM package** 2](#_Toc126599541)

[**Disallowed transitions in the Markov multi-state model** 3](#_Toc126599542)

[**Details of the STOP model** 3](#_Toc126599543)

[**Validation aims** 4](#_Toc126599544)

[**Supplemental references** 6](#_Toc126599545)

[**Supplemental tables and figures** 7](#_Toc126599546)

[**Table A.** 7](#_Toc126599547)

[**Table B** 9](#_Toc126599548)

[**Table C** 10](#_Toc126599549)

[**Fig A** 11](#_Toc126599550)

[**Fig B** 12](#_Toc126599551)

[**Fig C.** 13](#_Toc126599552)

# **Supplemental methods**

## **Adding survey weights to the MSM package**

By default, the R “msm” package estimates transition rates via maximum likelihood estimation.[1] An optimization algorithm accepts a starting set of transition rate estimates, calculates the model’s log-likelihood for those estimates, and iteratively adjusts the estimates and recalculates the log-likelihood until arriving at a solution: a set of transition rate estimates that produce the maximum log-likelihood value.[2] Annual transition probabilities are subsequently calculated from final transition rates.[1] Our R code for *weighted* maximum likelihood estimation pauses the optimization algorithm during every iteration, calculates each Population Assessment of Tobacco and Health (PATH) survey participant’s individual contribution to the model log-likelihood, multiplies by the participant’s longitudinal survey weights, and sums the result to estimate the overall model log-likelihood for the transition rate estimates. We estimate weighted transition probabilities using the default msm package function, substituting weighted transition rates for the default unweighted rates.

Conceptually, this is identical to a participant’s responses being duplicated in the survey $w_{i}$ times, where $w_{i}$ is the value of that participant’s longitudinal survey weight. This approach is appropriate for calculating point estimates of transition rates, but not for calculating their variances. We calculated weighted variances by fitting 100 separate models using each of the 100 PATH-provided replicate-weights and following *Equation 1)*, as recommended by the PATH User Guide.[3]

$v\left( \hat{\theta} \right)= 0.020408* \sum_{g=1}^{100} \left( \hat{\theta}_{(g)}- \hat{\theta} \right)^{2}$ Equation 1)

where $\theta$is the statistic of interest: in this case a transition rate, an annual first or cumulative transition probability, a rate ratio, or a difference in annual first or cumulative transition probabilities; $\thetâ$ is the estimate of $\theta$ calculated using the full-sample longitudinal survey weight; $v(\hat{\theta})$ is the variance of $\thetâ$; and $\hat{\theta}_{(g)}$ is the estimate of $\theta$using the $g$-th replicate weight.

To reduce computational requirements, we used unweighted transition rates as estimate starting values and limited the optimization algorithm to 25,000 iterations. Fig A shows the convergence of transition rate estimates with respect to iteration of the weighted maximum likelihood algorithm.

**Disallowed transitions in the Markov multi-state model**

We applied rules when determining which transitions would be disallowed in the Markov multi-state model (Fig 2 in the main paper). First, those who were “never users” of a particular product (never smokers or never e-cigarette [e-cig] users) could not transition directly to “former use” of that product. Second, “former users” and “current users” of a particular product could not transition to “never use” of that product.

**Details of the STOP model**

The original version of the Simulation of Tobacco and Nicotine Outcomes and Policy (STOP) microsimulation model included current, former, and never cigarette smoking states but did not include e-cig or dual use states.[4] We expanded the STOP model to include the same nine smoking and e-cig use states and 27 state transitions that were specified in the continuous time Markov multi-state model. STOP transitions, however, are divided into three types: start, quit, and relapse (Fig B). Monthly first transition probabilities are specified for each of the 27 state-transitions, as the STOP model only allows one transition per month. Start and quit probabilities are further stratified by age and sex, and relapse probabilities are further stratified by age only.

STOP is stochastic—it uses computer random number generation to simulate a cohort of patients passing through simulated health states with varying probabilities—and uses a monthly time step. It contains additional modules not utilized in this validation that incorporate chronic disease incidence and complications, mortality, and smoking and e-cig policy interventions.

When the time-variant relapse module of STOP is activated, relapse rates decay exponentially over time according to the equation $Ce^{-0.33t},$ where $t$ is the number of months since the participant’s last quit attempt and $C$ is the initial relapse rate.[4] The value of $C$ is set such that the overall probability of relapse in the first year after a cessation attempt is equal between static relapse and time-variant relapse simulations.

## **Validation aims**

In accordance with published guidelines on good practices in model validation, we performed face validation and internal validation.[5] STOP does not model birth or other migration into the cohort, so comparison targets needed to come from a longitudinal study. Due to limited availability of external longitudinal data on smoking and e-cig use, we validated STOP model output with the PATH data. At the time of writing, PATH was the only publicly available longitudinal dataset on smoking and e-cig use status in the US, and thus we used PATH Waves 3-4 smoking and e-cig use prevalence data to validate the behavioral transition parameters derived from PATH Waves 1-4.5. We demonstrated that behavioral transition estimates generated by our Markov multi-state model, when applied as behavioral transition parameters in a microsimulation model, accurately predict prevalence outcomes.

We considered independent external validation with cross-sectional datasets such as the National Youth Tobacco Survey (NYTS) and the National Health Interview Survey (NHIS). However, NYTS and NHIS lack details about smoking and e-cig relapse, an important component of the STOP microsimulation model and the multi-state Markov model that can only be captured by longitudinal data such as PATH. Additionally, questions about tobacco smoking and e-cig use differ across studies, complicating comparisons especially of dual use states.

In future analyses that aim to project future outcomes or to inform public health decisions and policy, STOP model parameters can be calibrated to fit data from cohorts of interest.

**Supplemental references**

1. Jackson C. Multi-state modelling with R: the msm package. The Comprehensive R Archive Network; Available: https://cran.r-project.org/web/packages/msm/vignettes/msm-manual.pdf

2. Myung IJ. Tutorial on maximum likelihood estimation. Journal of Mathematical Psychology. 2003;47: 90–100. doi:10.1016/S0022-2496(02)00028-7

3. Westat Corporation. Population Assessment of Tobacco and Health (PATH) Study [United ICPSR 37786 States] Special Collection Public-Use Files. United States National Institute on Drug Abuse and United States Center for Tobacco Products; Available: https://www.icpsr.umich.edu/web/NAHDAP/studies/37786/datadocumentation

4. Reddy KP, Bulteel AJB, Levy DE, Torola P, Hyle EP, Hou T, et al. Novel microsimulation model of tobacco use behaviours and outcomes: calibration and validation in a US population. BMJ Open. 2020;10: e032579. doi:10.1136/bmjopen-2019-032579

5. Eddy DM, Hollingworth W, Caro JJ, Tsevat J, McDonald KM, Wong JB, et al. Model transparency and validation: a report of the ISPOR-SMDM Modeling Good Research Practices Task Force-7. Med Decis Making. 2012;32: 733–743. doi:10.1177/0272989X12454579

# **Supplemental tables and figures**

| **Table A.** Included PATH participants by survey wave, age, sex, and smoking and e-cigarette use state, n (weighted %). | | | | | | | | | | |
| --- | --- | --- | --- | --- | --- | --- | --- | --- | --- | --- |
|  |  | Smoking and E-Cig Use State | | | | | | | | |
| Wave | Covariate | CSCE | CSFE | CSNE | FSCE | FSFE | FSNE | NSCE | NSFE | NSNE |
|  | *Age* |  |  |  |  |  |  |  |  |  |
|  | 12 to 17 years old | 0* | 0* | 131 (1.8%) | 0* | 0* | 17 (0.2%) | 0* | 0* | 7,679 (97.9%) |
|  | 18 to 24 years old | 168 (2.3%) | 81 (1.1%) | 1,211 (16.3%) | 37 (0.5%) | 29 (0.4%) | 247 (3.9%) | 78 (1.1%) | 31 (0.4%) | 3,755 (74.0%) |
| 1 | 25 to 44 years old | 295 (2.3%) | 115 (0.9%) | 2,589 (20.1%) | 104 (0.8%) | 56 (0.4%) | 991 (13.5%) | 33 (0.3%) | 11 (0.1%) | 3,131 (61.6%) |
|  | 45 or more years old | 174 (0.9%) | 67 (0.3%) | 2,567 (13.9%) | 94 (0.5%) | 36 (0.2%) | 1,712 (28.6%) | 33 (0.1%) | 12 (0.1%) | 3,026 (55.4%) |
|  | *Sex* |  |  |  |  |  |  |  |  |  |
|  | Female | 319 (1.3%) | 132 (0.5%) | 3,326 (13.4%) | 134 (0.6%) | 64 (0.3%) | 1,375 (16.9%) | 71 (0.2%) | 27 (0.1%) | 9,174 (66.7%) |
|  | Male | 318 (1.6%) | 131 (0.7%) | 3,172 (17.1%) | 101 (0.6%) | 57 (0.3%) | 1,592 (19.9%) | 73 (0.3%) | 27 (0.1%) | 8,417 (59.4%) |
|  | *Age* |  |  |  |  |  |  |  |  |  |
|  | 12 to 17 years old | 34 (0.4%) | 9 (0.1%) | 84 (1.1%) | 0 | 7 (0.1%) | 27 (0.4%) | 79 (1.1%) | 58 (0.7%) | 7,894 (96.1%) |
|  | 18 to 24 years old | 259 (3.4%) | 182 (2.5%) | 988 (13.0%) | 56 (0.8%) | 63 (0.9%) | 302 (4.3%) | 154 (2.1%) | 113 (1.6%) | 4,193 (71.4%) |
| 2 | 25 to 44 years old | 415 (3.2%) | 336 (2.5%) | 2,370 (18.3%) | 189 (1.5%) | 108 (0.8%) | 1,168 (14.4%) | 37 (0.3%) | 35 (0.3%) | 3,156 (58.9%) |
|  | 45 or more years old | 240 (1.2%) | 210 (1.1%) | 2,591 (13.9%) | 121 (0.7%) | 76 (0.4%) | 2,001 (30.8%) | 18 (0.1%) | 8 (0.0%) | 2,770 (51.9%) |
|  | *Sex* |  |  |  |  |  |  |  |  |  |
|  | Female | 467 (1.8%) | 385 (1.4%) | 3,087 (12.5%) | 175 (0.7%) | 124 (0.5%) | 1,634 (18.3%) | 120 (0.4%) | 82 (0.2%) | 9,450 (64.1%) |
|  | Male | 481 (2.3%) | 352 (1.8%) | 2,946 (15.8%) | 191 (1.1%) | 130 (0.6%) | 1,864 (21.4%) | 168 (0.6%) | 132 (0.5%) | 8,563 (55.9%) |
|  | *Age* |  |  |  |  |  |  |  |  |  |
|  | 12 to 17 years old | 30 (0.4%) | 15 (0.2%) | 46 (0.6%) | 4 (0.1%) | 12 (0.1%) | 17 (0.2%) | 97 (1.2%) | 74 (0.9%) | 7,939 (96.2%) |
|  | 18 to 24 years old | 239 (3.6%) | 262 (4.1%) | 634 (9.4%) | 73 (1.2%) | 97 (1.5%) | 215 (3.7%) | 185 (2.6%) | 199 (2.9%) | 4,125 (71.1%) |
| 3 | 25 to 44 years old | 363 (3.2%) | 559 (4.7%) | 1,784 (15.7%) | 189 (1.7%) | 227 (1.9%) | 1,043 (14.7%) | 44 (0.3%) | 67 (0.5%) | 2,745 (57.3%) |
|  | 45 or more years old | 212 (1.2%) | 340 (1.8%) | 2,117 (12.9%) | 152 (1.0%) | 111 (0.6%) | 1,961 (34.3%) | 11 (0.1%) | 12 (0.1%) | 2,216 (48.1%) |
|  | *Sex* |  |  |  |  |  |  |  |  |  |
|  | Female | 416 (1.8%) | 602 (2.4%) | 2,370 (11.2%) | 198 (1.0%) | 202 (0.9%) | 1,472 (19.5%) | 130 (0.4%) | 152 (0.4%) | 8,859 (62.4%) |
|  | Male | 428 (2.3%) | 574 (3.2%) | 2,211 (13.3%) | 220 (1.3%) | 245 (1.3%) | 1,764 (23.7%) | 207 (0.7%) | 200 (0.8%) | 8,166 (53.4%) |

| **Table A, Continued.** | | | | | | | | | | |
| --- | --- | --- | --- | --- | --- | --- | --- | --- | --- | --- |
|  |  | Smoking and E-Cig Use State | | | | | | | | |
| Wave | Covariate | CSCE | CSFE | CSNE | FSCE | FSFE | FSNE | NSCE | NSFE | NSNE |
|  | *Age* |  |  |  |  |  |  |  |  |  |
|  | 12 to 17 years old | 14 (0.2%) | 12 (0.2%) | 23 (0.4%) | 4 (0.0%) | 4 (0.1%) | 12 (0.2%) | 84 (1.1%) | 78 (1.0%) | 7,711 (97.0%) |
|  | 18 to 24 years old | 242 (3.3%) | 294 (4.3%) | 554 (7.5%) | 88 (1.4%) | 121 (1.8%) | 218 (3.3%) | 203 (2.7%) | 286 (3.8%) | 4,986 (71.9%) |
| 4 | 25 to 44 years old | 381 (3.1%) | 747 (6.1%) | 1,674 (14.3%) | 184 (1.7%) | 282 (2.4%) | 1,127 (14.8%) | 47 (0.4%) | 96 (0.7%) | 2,913 (56.5%) |
|  | 45 or more years old | 200 (1.1%) | 443 (2.3%) | 2,079 (12.3%) | 153 (0.9%) | 161 (1.0%) | 2,095 (35.0%) | 10 (0.1%) | 16 (0.1%) | 2,244 (47.1%) |
|  | *Sex* |  |  |  |  |  |  |  |  |  |
|  | Female | 408 (1.7%) | 761 (3.1%) | 2,230 (10.3%) | 197 (0.9%) | 263 (1.2%) | 1,586 (20.2%) | 127 (0.4%) | 210 (0.6%) | 9,265 (61.7%) |
|  | Male | 429 (2.1%) | 735 (4.0%) | 2,100 (12.4%) | 232 (1.3%) | 305 (1.7%) | 1,866 (24.2%) | 217 (0.8%) | 266 (0.9%) | 8,589 (52.6%) |
|  | *Sex* |  |  |  |  |  |  |  |  |  |
| 4.5 | Female | 14 (0.4%) | 4 (0.1%) | 19 (0.5%) | 4 (0.1%) | 0 | 5 (0.2%) | 95 (2.6%) | 21 (0.6%) | 3,605 (95.4%) |
|  | Male | 15 (0.4%) | 13 (0.3%) | 18 (0.5%) | 3 (0.1%) | 4 (0.2%) | 7 (0.2%) | 109 (2.8%) | 52 (1.3%) | 3,820 (94.2%) |
| NSNE = Never Smoker, Never E-Cig User NSFE = Never Smoker, Former E-Cig User NSCE = Never Smoker, Current E-Cig User … CSCE = Current Smoker, Current E-Cig User  PATH = Population Assessment of Tobacco and Health Study. *Current and former e-cig use could not be ascertained among Wave 1 youth due to limitations in the PATH questionnaire | | | | | | | | | | |

| **Table B.** Number of observed transitions between states of smoking and e-cigarette use among youth and adults in PATH Waves 1-4, unweighted. | | | | | | | | | | |
| --- | --- | --- | --- | --- | --- | --- | --- | --- | --- | --- |
|  |  | **To** |  |  |  |  |  |  |  |  |
|  | **From** | NSNE | NSFE | NSCE | FSNE | FSFE | FSCE | CSNE | CSFE | CSCE |
| **Youth** | NSNE | --- | 200 | 519 | 38 | 10 | 7 | 163 | 8 | 43 |
|  | NSFE | 0 | --- | 55 | 0 | 3 | 1 | 0 | 13 | 4 |
|  | NSCE | 0 | 86 | --- | 0 | 1 | 0 | 0 | 8 | 16 |
|  | FSNE | 0 | 0 | 0 | --- | 1 | 2 | 19 | 2 | 6 |
|  | FSFE | 0 | 0 | 0 | 0 | --- | 2 | 0 | 4 | 4 |
|  | FSCE | 0 | 0 | 0 | 0 | 0 | --- | 0 | 3 | 1 |
|  | CSNE | 0 | 0 | 0 | 37 | 3 | 2 | --- | 11 | 22 |
|  | CSFE | 0 | 0 | 0 | 0 | 4 | 2 | 0 | --- | 9 |
|  | CSCE | 0 | 0 | 0 | 0 | 5 | 3 | 0 | 18 | --- |
| **Adult** | NSNE | --- | 199 | 275 | 624 | 14 | 31 | 957 | 24 | 63 |
|  | NSFE | 0 | --- | 73 | 0 | 16 | 0 | 0 | 39 | 9 |
|  | NSCE | 0 | 243 | --- | 0 | 19 | 19 | 0 | 49 | 71 |
|  | FSNE | 0 | 0 | 0 | --- | 132 | 93 | 869 | 53 | 47 |
|  | FSFE | 0 | 0 | 0 | 0 | --- | 58 | 0 | 159 | 47 |
|  | FSCE | 0 | 0 | 0 | 0 | 181 | --- | 0 | 91 | 197 |
|  | CSNE | 0 | 0 | 0 | 1616 | 84 | 190 | --- | 599 | 859 |
|  | CSFE | 0 | 0 | 0 | 0 | 202 | 57 | 0 | --- | 382 |
|  | CSCE | 0 | 0 | 0 | 0 | 170 | 244 | 0 | 1126 | --- |
| NSNE = Never Smoker, Never E-Cig User NSFE = Never Smoker, Former E-Cig User NSCE = Never Smoker, Current E-Cig User … CSCE = Current Smoker, Current E-Cig User  PATH = Population Assessment of Tobacco and Health Study.  This table shows the number of transitions observed in PATH Waves 1-4. Accordingly, some transitions (e.g., NSNE to NSFE) are shown here but are disallowed in the multi-state model. The multi-state model interprets these as multiple sequential transitions between Waves, one or more of which are not explicitly observed. The numbers in this table are empirically observed transitions and are therefore not weighted. They are most appropriately interpreted to understand the sample size of each transition and should not be used to compare the frequency of transitions. | | | | | | | | | | |

| **Table C**. Validation of transition hazard rate estimates in the Simulation of Tobacco and Nicotine Outcomes and Policy (STOP) microsimulation model. | | | | | | |
| --- | --- | --- | --- | --- | --- | --- |
| Wave | Smoking and  E-Cig Use State | Static Relapse Projection of Prevalence | Time-Variant Relapse Projection of Prevalence | Empirical Estimate of Prevalence from PATH | Static Relapse Error:  Estimate (95% CI) | Time-Variant Relapse Error:  Estimate (95% CI) |
|  | NSNE | 57.9% | 57.9% | 57.5% | 0.4% (-0.8 to 1.6%) | 0.4% (-0.8 to 1.6%) |
|  | NSFE | 0.5% | 0.5% | 0.6% | -0.1% (-0.2 to 0.0%) | -0.1% (-0.2 to 0.0%) |
|  | NSCE | 0.8% | 0.8% | 0.6% | **0.2% (0.1 to 0.3%)** | **0.2% (0.1 to 0.4%)** |
|  | FSNE | 20.9% | 20.1% | 21.8% | -0.9% (-1.9 to 0.0%) | **-1.7% (-2.7 to -0.7%)** |
| 3 | FSFE | 1.0% | 0.9% | 1.1% | -0.1% (-0.2 to 0.0%) | **-0.2% (-0.4 to -0.1%)** |
|  | FSCE | 1.2% | 1.2% | 1.2% | 0.1% (-0.1 to 0.2%) | 0.1% (-0.1 to 0.3%) |
|  | CSNE | 12.9% | 13.4% | 12.5% | 0.5% (-0.2 to 1.1%) | **1.0% (0.3 to 1.7%)** |
|  | CSFE | 2.3% | 2.5% | 2.8% | **-0.6% (-0.8 to -0.4%)** | **-0.4% (-0.6 to -0.2%)** |
|  | CSCE | 2.6% | 2.8% | 2.0% | **0.5% (0.3 to 0.7%)** | **0.7% (0.5 to 0.9%)** |
|  | NSNE | 56.1% | 56.1% | 56.0% | 0.1% (-1.2 to 1.5%) | 0.1% (-1.2 to 1.5%) |
|  | NSFE | 0.9% | 0.9% | 0.8% | 0.1% (0.0 to 0.3%) | 0.1% (0.0 to 0.3%) |
|  | NSCE | 1.1% | 1.0% | 0.6% | **0.5% (0.3 to 0.7%)** | **0.4% (0.3 to 0.6%)** |
|  | FSNE | 20.6% | 22.4% | 22.7% | **-2.1% (-3.2 to -1.0%)** | -0.3% (-1.3 to 0.7%) |
| 4 | FSFE | 1.5% | 1.5% | 1.4% | 0.0% (-0.2 to 0.2%) | 0.1% (-0.1 to 0.3%) |
|  | FSCE | 1.5% | 1.4% | 1.2% | **0.4% (0.2 to 0.6%)** | 0.3% (0.0 to 0.5%) |
|  | CSNE | 12.2% | 10.5% | 11.7% | 0.5% (-0.4 to 1.3%) | **-1.2% (-1.9 to -0.5%)** |
|  | CSFE | 3.2% | 3.1% | 3.6% | **-0.5% (-0.7 to -0.2%)** | **-0.5% (-0.8 to -0.2%)** |
|  | CSCE | 3.1% | 2.9% | 2.0% | **1.1% (0.9 to 1.3%)** | **0.9% (0.7 to 1.1%)** |
| Static relapse rate STOP projections of smoking and e-cig use prevalence are compared with time-variant relapse rate STOP projections and empirical estimates from the Population Assessment of Tobacco and Health (PATH) Study. Static relapse STOP simulations have a constant relapse rate with respect to the number of months of abstinence, while time-variant STOP simulations have a relapse rate that decays exponentially with respect to the number of months of abstinence. Error is measured as the difference between the model-projected prevalence of each smoking and e-cig use state and the corresponding empirical prevalence in PATH.  NSNE = Never Smoker, Never E-Cig User NSFE = Never Smoker, Former E-Cig User NSCE = Never Smoker, Current E-Cig User … CSCE = Current Smoker, Current E-Cig User  **Bold** indicates that the p value of the error is < 0.05. | | | | | | |

**
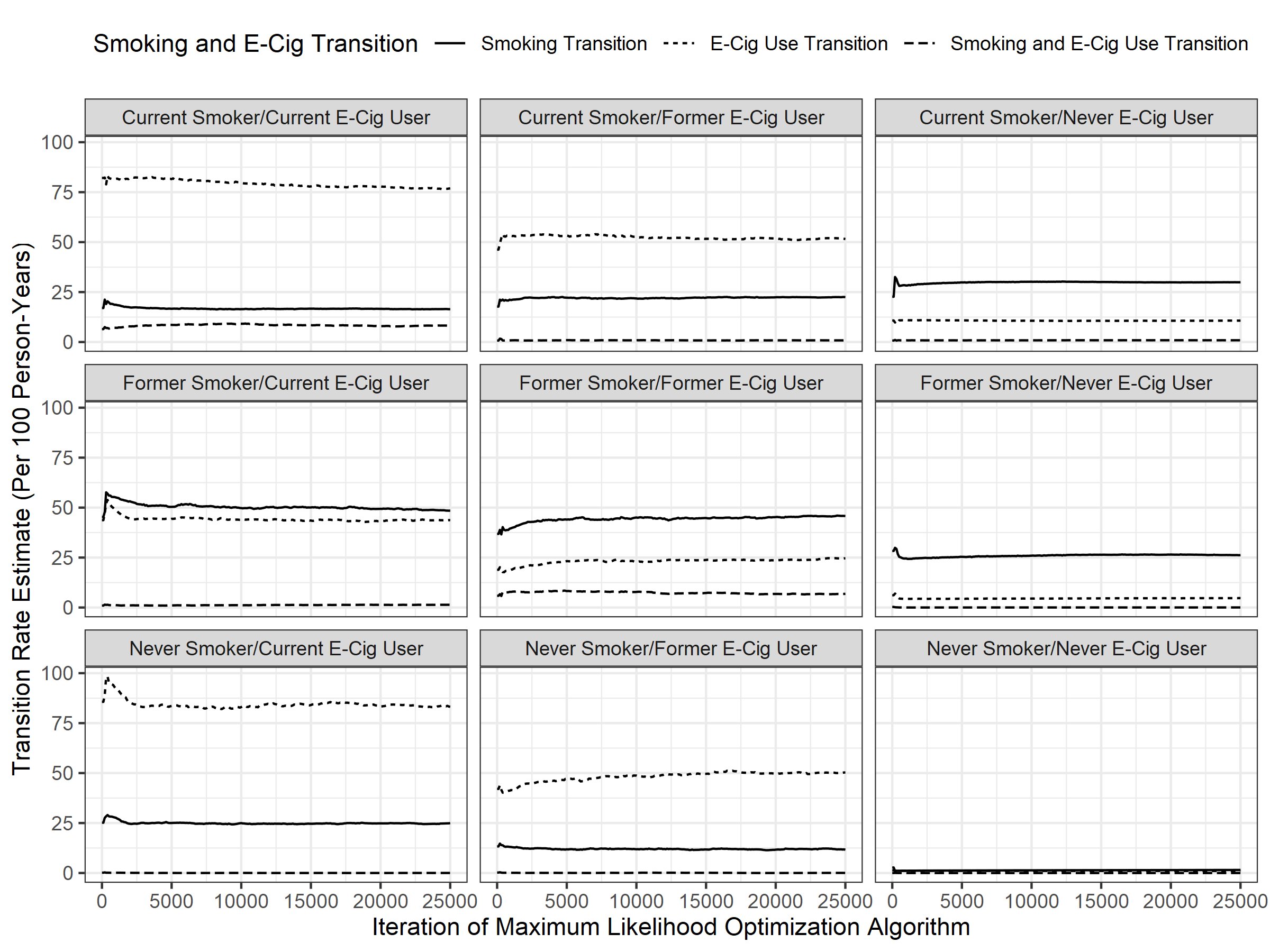
****Fig A.** Changes in transition rate estimates with respect to iteration of the weighted maximum likelihood optimization algorithm.

Transition rate estimates for each of the three possible transitions out of every smoking and e-cig use state are extracted from the continuous time Markov multi-state model every 100 iterations of the maximum likelihood optimization algorithm. The visualized rates are an average of age- and sex-specific rates, according to their weighted prevalence in the Population Assessment of Tobacco and Health (PATH) data. Estimate convergence, as indicated by a relatively flat line, occurs before 25,000 iterations in all transitions.

**Fig B.** Monthly transitions categorized by type in the Simulation of Tobacco and Nicotine Outcomes and Policy (STOP) microsimulation model.


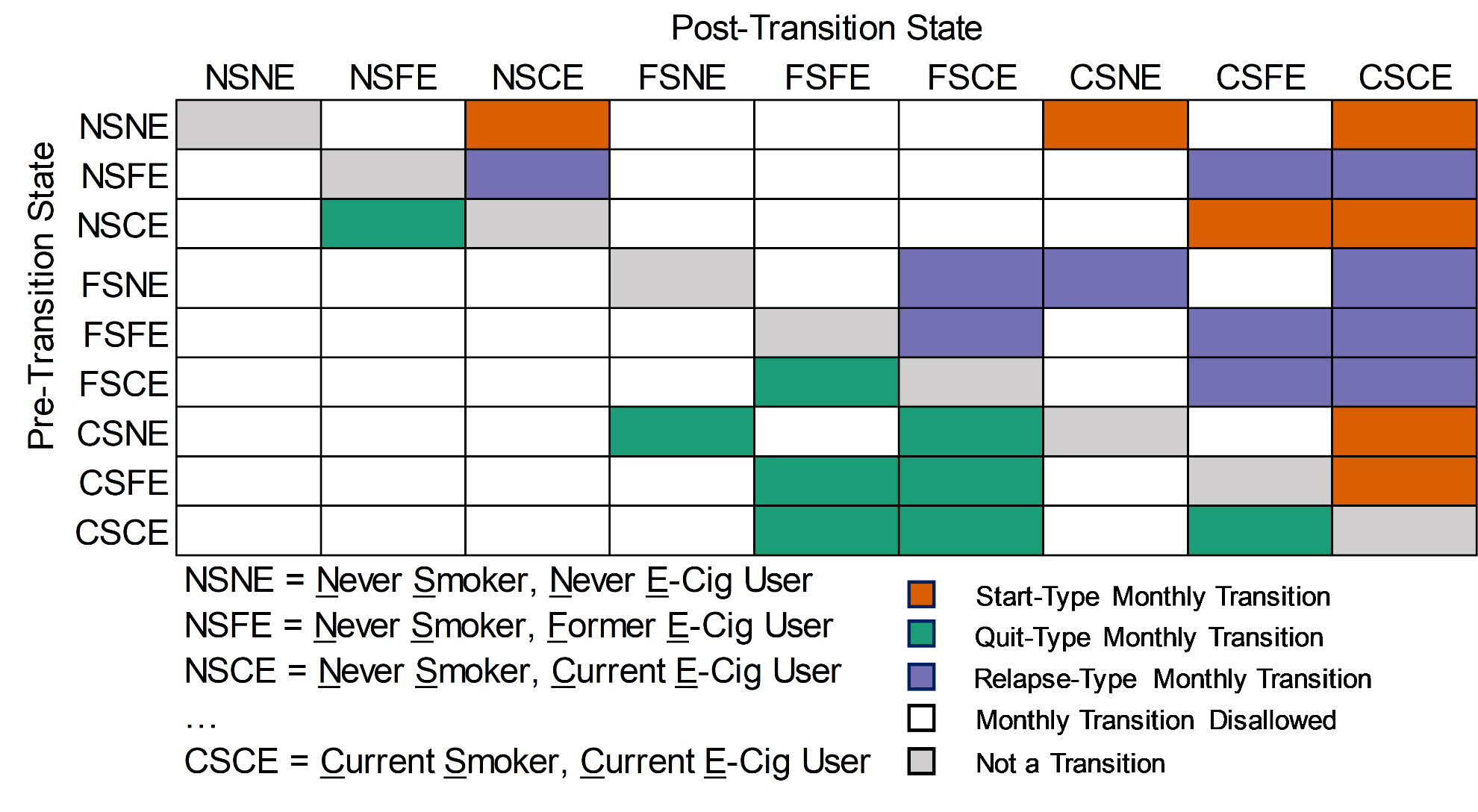


The STOP model allows no more than one transition to occur each month. Monthly transitions from the indicated pre-transition smoking and e-cig use state (row) to the indicated post-transition state (column) are categorized by type: start (red), quit (green), and relapse (purple). Start and quit transitions are stratified by age, sex, and current smoking and e-cig use state, but are constant with respect to time. Relapse transitions are stratified by age and current smoking and e-cig use state and have the option to decay exponentially with respect to duration of abstinence. Disallowed instantaneous transitions are in white.

**
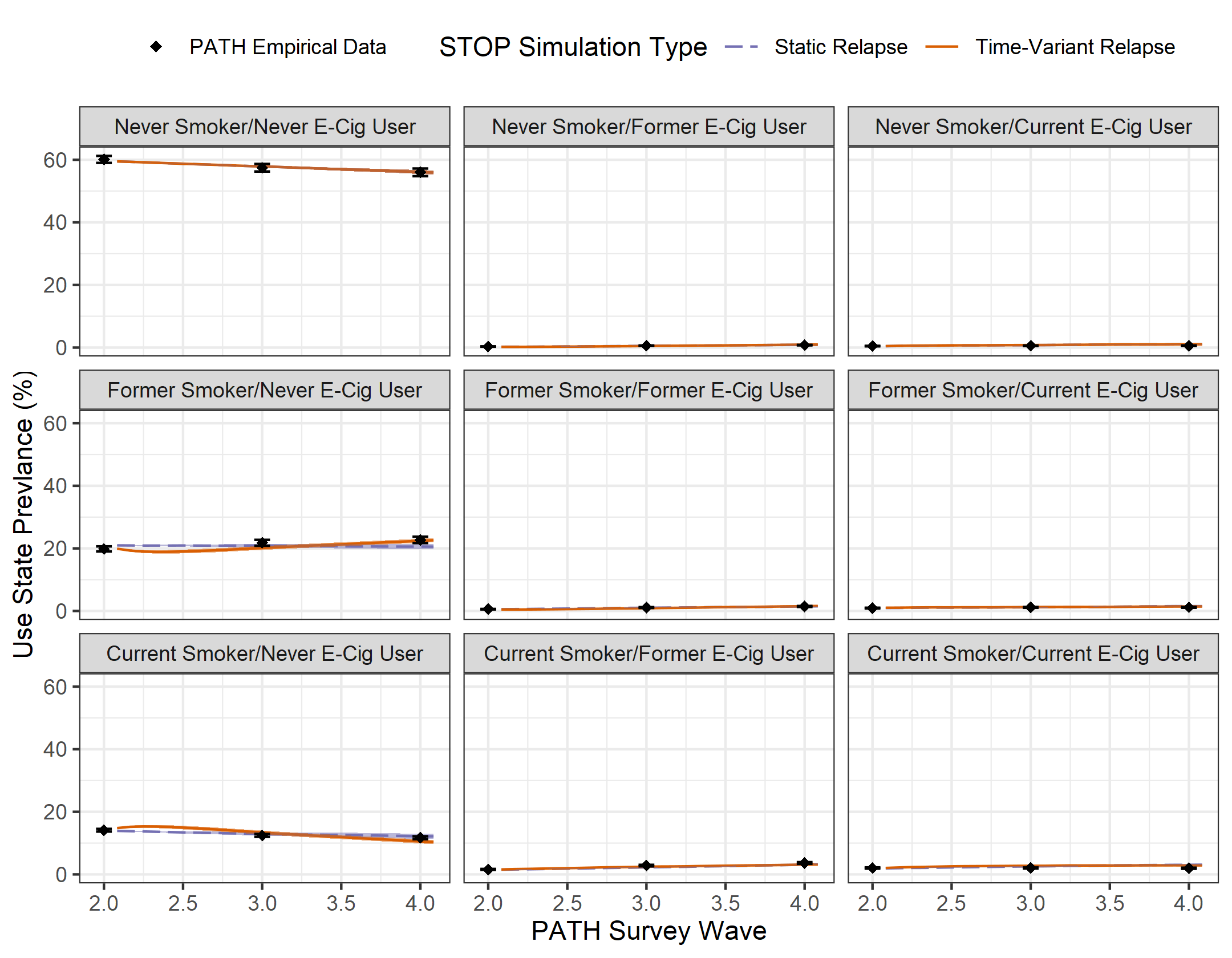
****Fig C.** Validation of the Simulation of Tobacco and Nicotine Outcomes and Policy (STOP) microsimulation: prevalence of each Markov smoking and e-cig use status.

The STOP model is parametrized with Markov multi-state model-estimated smoking and e-cig transition hazard rates, along with Population Assessment of Tobacco and Health (PATH) Wave 2 smoking and e-cig use prevalence. The static relapse simulation has constant relapse rates with respect to duration of abstinence, while the time-variant relapse simulation allows relapse rates among former smokers and former e-cig users to decay exponentially according to their durations of abstinence. STOP-projected 12-month and 24-month estimates of prevalence of smoking and e-cig use are compared with empirical data from PATH Waves 3 and 4. In some plots, the static relapse simulation line and the time-variant relapse simulation line are essentially superimposed. 95% confidence intervals are shown as shaded blue and orange segments for STOP static relapse and time-variant relapse simulations, respectively, and error bars for PATH empirical estimates. Confidence intervals are narrow enough to be nearly invisible in some sub-plots.
